# Is It Possible to Reshape the Body and Tone It at the Same Time? Schwarzy: The New Technology for Body Sculpting

**DOI:** 10.3390/bioengineering9070284

**Published:** 2022-06-28

**Authors:** Francesca Negosanti, Giovanni Cannarozzo, Tiziano Zingoni, Alessandro Leone, Irene Fusco

**Affiliations:** 1 Dermatologic Center, 40141 Bologna, Italy; francesca.negosanti@gmail.com; 2 Lasers in Dermatology Unit, Department of Dermatology, University of Rome Tor Vergata, 00133 Rome, Italy; drcannarozzo@gmail.com (G.C.); tizingo@tin.it (T.Z.); 3 Dermatos Center, Via Vestina 216, 65015 Montesilvano, Italy; alessandroleone85@gmail.com; 4 Department of Biology, University of Florence, 50121 Florence, Italy

**Keywords:** muscle tone, body sculpting, Flat Magnetic Stimulation

## Abstract

*Background and Objective:* In recent years, a strong desire for slimmer and healthier-looking bodies has grown in the population. The aim of this study was to evaluate the effectiveness and safeness of the new technology Flat Magnetic Stimulation for buttock and abdomen remodeling in athletic subjects. *Methods:* A total of 49 patients (31 females and 18 males) were enrolled. Patients’ digital photos and buttocks/abdomen circumference measurements were taken to assess and monitor the effectiveness of treatment on muscle firming. The level of patient satisfaction was evaluated by a questionnaire based on a seven point Likert scale. Average scores were calculated at a 1-month follow-up (FU). *Results:* A significant increase in the buttocks’ mean circumference from 85.5 ± 0.7 cm to 88.5 ± 0.7 cm (*p* < 0.05) and in the abdomens’ mean circumference from 76.5 ± 9.19 cm to 78 ± 9.89 cm (*p* < 0.05) was observed 1 month after the last treatment. All subjects reported that their buttocks and abdomens felt more lift and toned. The average abdomen and buttocks satisfaction scores improve significantly at 1-month FU. *Conclusions:* Our data show that FMS treatment could be used as an effective mechanism for muscle toning.

## 1. Introduction

In recent years, a strong desire for slimmer and healthier-looking bodies has grown in the population. For health promotion, the quantitative assessment of skeletal muscle mass and fat mass was important.

Findings from epidemiological studies have shown that visceral adipose tissue is an independent risk marker of cardiovascular and metabolic morbidity and mortality. Emerging evidence also suggests that ectopic fat deposition might contribute to increased atherosclerosis and cardiometabolic risk [1].

Therefore, maximizing muscle mass is of fundamental importance for all those populations associated with sport and health. In the initial stages of resistance training, in untrained subjects, muscle hypertrophy is practically non-existent and most of the strength gains derive from neural adaptations [2].

Factors such as age, genetic background, or gender cause hypertrophy in response to a training protocol, affecting both the rate and amount of lean muscle mass [3]. Additionally, as training experience is gained it becomes more and more difficult to increase lean muscle mass by having the right routine design.

Therefore, the quantitative evaluation of fat and skeletal muscle and its strengthening is very important to maintain good health and improve the aesthetic appearance of training subjects.

Muscle hypertrophy is distinct and separate from muscle hyperplasia [4], in which an increase in the diameter of the individual fibers and therefore an increase in the area of the muscular cross section can be observed [5].

The high risk of onset, long downtime and financial costs associated with surgical procedures, such as liposuction (effective in removing large amounts of fat but associated with severe side effects such as edema, postoperative pain, scarring and various infections), for body remodeling have led to the development of numerous non-invasive techniques.

Current surgical and non-invasive body shaping procedures often require patients with already good BMI for safe and successful treatment.

Therefore, known body contouring procedures, such as radiofrequency, cryolipolysis or high intensity focused ultrasound (HIFU), are not suitable for a lot of patients, especially those with lower BMI [6].

For a long time, exercise was the only option for toning the muscle; despite the fact that several procedures which use electrical stimulation for muscle strengthening have been introduced [7,8], their efficacy has been considered controversial.

The electrical stimulation is able to depolarize the motor neurons, thus helping to contract the muscles [9]. Most of the energy is concentrated, during electrical stimulation, on body surface, leading to skin overheating and therefore the risk of burns [10].

In addition, nociceptors are activated, and, at higher intensities, they make the procedure highly painful, [11] thus limiting the use of efficient settings. For these reasons non-invasive body remodeling is today the area that is growing and evolving fastest in aesthetic medicine [12].

It is hypothesized that all these techniques determine the release of triglycerides from fat cells and cause the necrosis or apoptosis of the targeted adipocytes. Other techniques have been studied and these include extracorporeal shock wave therapy (ECST), topical creams, acoustic wave therapy and mesotherapy. Nevertheless, the safeness and effectiveness of these techniques has not been established [6].

Magnetic Resonance Modeling with High Intensity Focused Electromagnetic Technology is the latest advancement in non-invasive body shaping. Magnetic stimulation has been used to effectively treat various medical conditions, such as urogynecological, neuropsychiatric and musculoskeletal disorders [13,14]. Compared to other non-invasive therapies for fat reduction on the market, MRI has the ability to improve muscle strength, thickness and tone. In 2018, the FDA approved the high intensity focused electromagnetic field for buttock and abdomen remodeling [15].

Electromagnetic stimulation is able to overcome the disadvantages of electrical stimulation as it penetrates deeper into the tissue without the risk of pain or burns [11,16]; this procedure can induce supramaximal involuntary contractions that cause muscle growth and also a reduction of subcutaneous fat in (but not limited to) patients with low body mass index. The procedure, thanks to this dual aspect, allows professionals to agree to the entire request of the patient and offers a truly innovative approach to body contouring.

In this research we have analyzed the effectiveness and safeness of Schwarzy (DEKA MELA, Calenzano, Italy) on the strengthening and improvement of muscle tone in training subjects. This is a new approach to body shaping [17].

This system uses FMS (Flat Magnetic Stimulation) technology to strengthening muscle mass through neuromuscular stimulation. Magnetic stimulation is able to activate the motor neurons at depth by causing large and deep muscle contractions since the magnetic field has the ability to penetrate up to 7 cm depth [18].

## 2. Materials and Methods

### 2.1. Patient Selection

A total of 49 patients (31 females and 18 males) with a BMI of 22–25 kg m^2^ and with an age ranging from 30 to 65 for female subjects and an age ranging from 40 to 65 for male subjects, were enrolled at the Villa Bella center, Bologna, Italy.

All subjects were healthy and undergoing training, with the desire to strengthen their muscles.

The exclusion criteria were: pregnant patients or patients with implanted electronic/metallic devices, cardiac pacemakers and/or any type of medical condition contraindicated for the use of the electromagnetic field, menstruation and subjects undergoing other treatments for body remodeling. No patients underwent caloric restriction during the entire treatment period. No subjects took any vitamins and supplements, and the female patients did not take any hormonal contraception.

### 2.2. Study Protocols

Schwarzy system represents a new approach to body sculpturing that, thanks to its non-invasive TOP FMS (Flat Magnetic Stimulation) technology, induces intense muscle contractions, stimulating motor neurons, not achievable through voluntary contractions. The FMS device has a circular coil positioned inside the applicator and at the level of which an alternating electric current is created, which in turn generates electromagnetic pulses with an intensity up to 2.5 Tesla.

This device shows advanced performance, thanks to its flat-type emission, which allows a more homogeneous distribution of the intensity that directly reaches the tissues in depth, without any skin damaging effects [17].

The device can act on different areas of the body thanks to its handpieces (flat, curve, ellipse) that adapt to the abdomen, buttocks, arms and legs, causing muscle contractions activated by electromagnetic energy.

Treatment duration ranged from 20 to 45 min, based on protocols. The technology uses three types of different protocols (aerobic, muscle definition and muscular strengthening) that consider both the current condition of the patient (training state) and their physical evolution, in terms of the improvement of the muscular state. The programs, therefore, are suggested for the resumption or strengthening of muscles from sedentary to fit patients.

Protocols have been programmed with active phases alternating with rest phases, allowing optimal muscle recovery of the patient and to avoid the onset of lactacidosis. These extreme conditions of muscular work are not reproducible either through the normal muscle contractions that take place in the gym and not with weight-lifting or functional training [17].

Eight treatment sessions were performed. The frequency of treatment was twice a week, leaving a minimum of two days between each session, in conformity with the ethical guidelines of the Declaration Helsinki (1975).

The treatment is comfortable as it does not involve dermal–epidermal interaction and therefore does not require any type of anesthesia; it is, above all, the presence of the handpieces and a liquid cooling system that guarantees good performance, avoiding the heating of the treated area and pain. Muscle shaping/strength protocol with an ellipse handpiece was performed on the abdomen of 25 patients in total (18 males and 7 females) and the muscle shaping/strengthening protocol with two flat handpieces was performed on the buttocks of 24 female patients in total. Each protocol is made up of 2 modules of increasing intensity, to be performed in chronological succession: 1—beginner step, to perform in the initial treatments of the series; 2—advanced step, to perform when well tolerated by subjects, to progress with the series of treatments.

To evaluate treatment effectiveness, buttocks and abdomen circumferences measurements were conducted.

Lateral and frontal digital photographs were executed before treatment and at a 1-month follow-up after the last treatment. To measure gluteus and abdomen circumferences, a flexible but inelastic anthropometric tape was used, considering a same reference point.

The level of patient satisfaction during treatment sessions was assessed by a questionnaire based on a 7 point Likert scale. The total possible scores ranged from 1 (very dissatisfied) to 7 (very satisfied). Average scores were calculated at baseline and at 1-month follow-up (FU).

A written patient consent form was released and archived.

### 2.3. Statistical Analysis

A paired Student’s *t* test was used, and statistical data were obtained with the SPSS (IBM Corp., New York, NY, USA) program, version 25.0 (IBM).

## 3. Results

**Figure 1:**
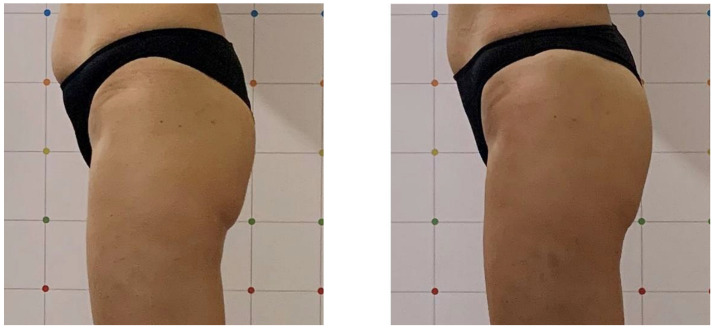
Left lateral view of a female patient before and 1 month after treatment. A good aesthetic improvement in buttocks muscle tone can be observed in the treated area.

**Figure 2:**
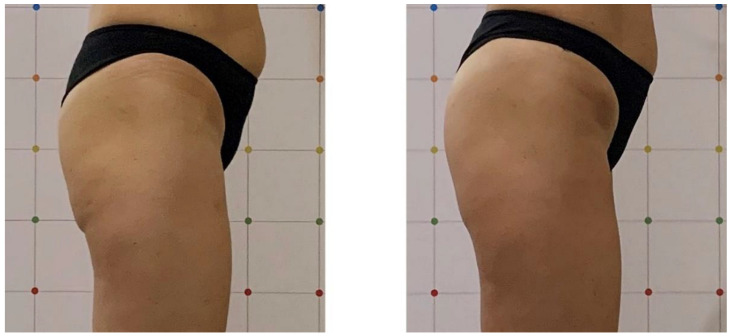
Right lateral view of a female patient before and 1 month after treatment. A good aesthetic improvement in buttocks muscle tone can be observed in the treated area.

**Figure 3:**
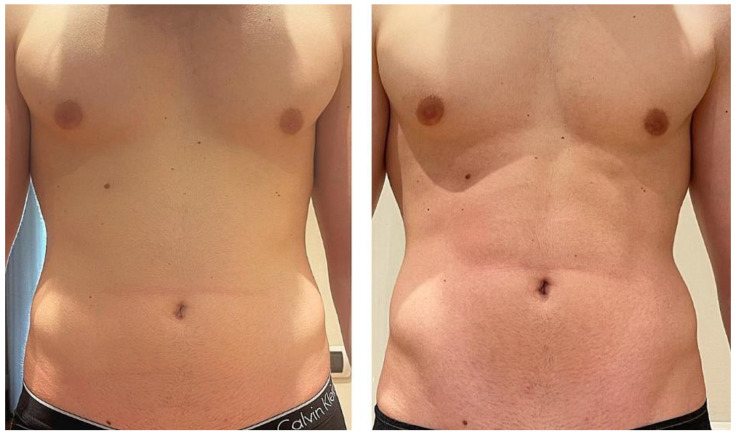
Abdominal frontal view of a male patient before and 1 month after treatment. A visible improvement in muscle tone can be observed.

**Figure 4:**
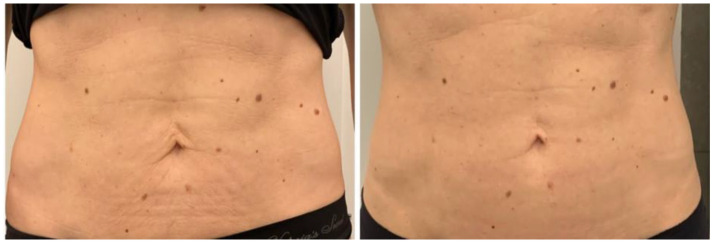
Abdominal frontal view of a female patient before and 1 month after treatment. A visible improvement in muscle tone can be observed.

All patients completed a course of treatments and were evaluated with a visit 1 month after treatment. After the 1-month follow-up, our results showed an evident muscle tonification; this device was able to reproduce the same metabolic effects by enhancing and firming the areas of interest. An aesthetic muscle improvement of the shape and volume of the treated area was shown by digital photographs. High treatment satisfaction was shown by all patients after each treatment sessions. All subjects reported that their buttocks and abdomens felt more lift and toned. The average abdomen satisfaction score at baseline was 12.8 ± 3.1 and increased at the 1-month (17.7 ± 2.6) follow-up (*p* < 0.01). The average buttocks satisfaction score at baseline was 10.5 ± 1.9 and increased at the 1-month (16.6 ± 2.7) follow-up (*p* < 0.01) (see Table 1 and Table 2). We observed a significant increase in the buttocks’ mean circumference (from 85.5 ± 0.7 cm to 88.5 ± 0.7 cm, *p* < 0.05) and in the abdomens’ mean circumference (from 76.5 ± 9.19 cm to 78 ± 9.89 cm, *p* < 0.05) 1 month after the last treatment (see Table 3 and Table 4). Digital photographs (Figure 1, Figure 2, Figure 3 and Figure 4) show good aesthetic results, with a visible improving of shape and volume of the treated areas and a reduction in muscle laxity. No adverse events were noted. We observed muscle fatigue as the only side effect that resolved within 48 h.

## 4. Discussion

Electromagnetic field devices, particularly the High Intensity Focused Electromagnetic Field (HIFEM), include the latest generation of dermatological technologies. The FDA permission for these technologies includes intended uses such as “stimulating neuromuscular tissue for muscle mass excitation in the legs or arms” and improving and strengthening abdominal tone [19,20].

Other studies on other possible applications, such as fat apoptosis and pelvic floor stimulation, are also ongoing [21]. Current data suggest that the physiological mechanism underlying magnetic stimulation is based on contractions that trigger intensive lipolysis within fat cells, leading to a large release of free fatty acids that damage the surrounding adipose tissue [22]. Damage to adipocytes causes apoptosis, as demonstrated in a study in which a 91.7% increase in the apoptotic ratio was observed in many histological samples [23]. This produces a desirable reduction in fat. The stress of rapid nerve discharge and contractions of muscle fibers also leads to compensatory muscle thickening.

A further advantage of magnetic stimulation at the muscular level is represented by a reduction in the distance between the large abdominal muscles.

This result was seen in 91% of patients, although recti diastasis was clinically not present. Complications following treatment with magnetic stimulation are minimal, in fact, the only side effect was a slight transient muscle pain in a small number of patients [24]. Jacob and Paskova [25] reported a 92% patient contentment 3 months after completing treatment regarding the abdomen.

In the literature it has been shown that magnetic stimulation is able to lift and tone the gluteal muscles; authors proved a significant improvement in buttocks aspect and a high grade of patient satisfaction [26].

The magnetic stimulation-induced muscle hypertrophy, fat reduction and reduction in abdominal separation has been observed in several studies [27,28,29]. Kent et al. [30], who investigated the effects of high-intensity focused electromagnetic technology for the induction of changes in abdominal muscles and abdominal subcutaneous fat, showed a significant patient subcutaneous fat reduction and the simultaneous thickening of the rectus abdominis muscle without reporting any sign of pain or discomfort.

This technology produced rectus muscle hypertrophy and a reduction in subcutaneous abdominal fat [17].

Clinical results obtained at a 1-month follow-up after the last treatment using the subject device, showed the tonification, enhancement and strengthening of the gluteal and abdominal muscles, with a reduction of localized adiposity. The treatment led to a significant increase in the patients’ buttock/abdomen circumferences.

The treatment is not painful, there are only intense and tolerable muscle contractions, it is totally non-invasive, takes place in maximum comfort and does not damage the dermo–epidermal tissue in the least.

**Figure 5:**
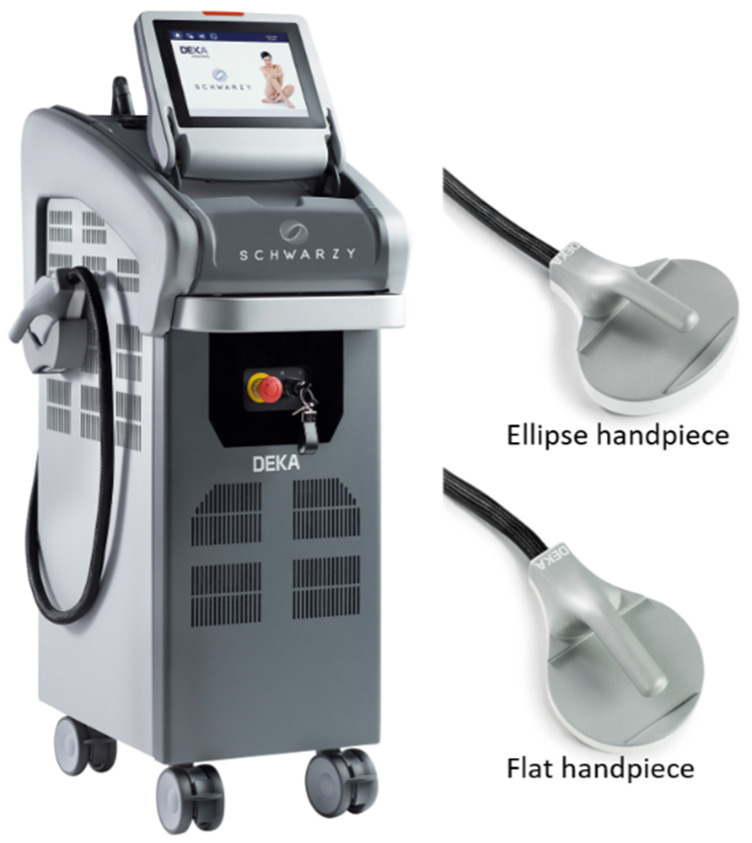
Representation of the Schwarzy system with two of its handpieces. Courtesy of DEKA MELA company, Calenzano, Italy.

The study subject device, with its handpieces, shown in Figure 5, adapts perfectly to the different areas of the body treated and transmits electromagnetic energy directly to the muscle tissue, contracting and releasing it.

This type of stimulation is superior to that achievable voluntarily by the user and it is already successfully used for body remodeling with excellent results [17,31].

Furthermore, a recent study published by Nisticò and colleagues [32] demonstrates how the combination of FMS and microwave treatment is safe and efficient for the treatment of abdominal subcutaneous fat and skin laxity, leading to a significant improvement in abdominal muscle tissue thickness, showing excellent results. Similarly, to our research, there are already recently published studies [33,34] in which waist circumference measurements after magnetic stimulation treatments sessions were used to assess patient’s muscle toning improvement with excellent results. In addition, in these studies, similar to our outcomes, original questionnaires were used to evaluate patient satisfaction concerning the improvement of their abdominal and buttocks area.

The benefits of the subject device include an increase in muscle tone and mass, maximum comfort, completely painless procedure, no recovery time, improvement of posture and the presence of pre-set and customized protocols.

Study results suggest that FMS technology is feasible for the aesthetic enhancements of the buttocks and abdomen utilizing a safe and comfortable treatment.

FMS technology is able to give a harmonious appearance to the buttocks and abdomen without inducing large and unnatural volumetric changes, as often happens with the use of surgical silicone implants, improving sporty appearance and postural aspect.

The subject device is very simple to use, and it does not require consumables for proper use.

The patients showed relatively high satisfaction during the treatment sessions.

### Study Limitations

Our future goal will be to increase the number of patients, also focusing on a greater number of male patients.

## 5. Conclusions

Our preliminary data show that TOP FMS represents a promising non-invasive technology for buttock and abdominal lifting/toning, in both active and inactive subjects.

## Figures and Tables

**Table 1:** Buttock satisfaction questionnaire results.

Question (Score Range, 1–7)	Baseline Mean	1 M FU Mean	Significance	Change
*Please rate your subjective perception of your buttock laxity/tightness*				
Total (*n* = 24)	2.8 ± 1.6	4.1 ± 0.9	(*p* < 0.01)	1.4
*Am I satisfied with the overall aesthetic appearance of my buttocks?*				
Total (*n* = 24)	2.4 ± 1.2	5.1 ± 1.4	(*p* < 0.01)	2.8
*I am satisfied with the shape of my buttocks?*				
Total (*n* = 24)	2.8 ± 1.6	3.9 ± 1.5	(*p* < 0.01)	1.1
*Do I feel confident about my buttocks area when wearing a swimsuit?*				
Total (*n* = 24)	2.6 ± 1.4	3.4 ± 1.3	(*p* < 0.05)	0.8
*Total score*	10.5 ± 1.9	16.6 ± 2.7	(*p* < 0.01)	6.0

**Table 2:** Abdomen satisfaction questionnaire results.

Question (Score Range, 1–7)	Baseline Mean	1 M FU Mean	Significance	Change
*Please rate your subjective perception of your abdomen laxity/tightness*				
Total (*n* = 25)	3.6 ± 1.3	4.1 ± 0.9	(*p* < 0.05)	0.6
*Am I satisfied with the overall aesthetic appearance of my abdomen?*				
Total (*n* = 25)	2.8 ± 1.6	5.4 ± 1.3	(*p* < 0.01)	2.6
*Am I satisfied with the shape of my abdomen?*				
Total (*n* = 25)	3.1 ± 1.6	4.2 ± 1.5	(*p* < 0.05)	1.1
*Do I feel confident about my abdomen area when wearing a swimsuit?*				
Total (*n* = 25)	3.4 ± 1.7	4.0 ± 1.3	(*p* < 0.05)	0.6
*Total score*	12.8 ± 3.1	17.7 ± 2.6	(*p* < 0.01)	4.9

**Table 3:** Mean buttock circumference.

Buttock Circumference	Mean ± SD [cm]	Significance
**Pretreatment**	85.5 ± 0.7	*p* < 0.05
**Posttreatment (1 M FU)**	88.5 ± 0.7	
**Change [cm]**	3.0	

**Table 4:** Mean abdomen circumference.

Abdomen Circumference	Mean ± SD [cm]	Significance
**Pretreatment**	76.5 (±9.19)	*p* < 0.05
**Posttreatment (1 M FU)**	78 (±9.89)	
**Change [cm]**	1.5	

## Data Availability

The data that support the findings of this study are available on request from the corresponding author (IF).
